# The Effects of Serotonergic Psychedelics on Neural Activity: A Meta-Analysis of Task-Based Functional Neuroimaging Studies

**DOI:** 10.1192/j.eurpsy.2023.1948

**Published:** 2023-07-19

**Authors:** J. H. Shepherd, C. Baten, A. Klassen, G. Zamora, S. Saravia, E. Pritchard, Z. Ali, S. K. Kahlon, K. Whitelock, F. A. Reyes, D. W. Hedges, J. P. Hamilton, M. D. Sacchet, C. H. Miller

**Affiliations:** 1 Department of Psychology, California State University, Fresno; 2 Department of Psychology, University of California, San Diego; 3 Department of Psychology, Brigham Young University, Provo, United States; 4 Department of Biomedical and Clinical Sciences, Linköping University, Linköping, Sweden; 5 Meditation Research Program, Department of Psychiatry, Massachusetts General Hospital, Harvard Medical School, Boston, United States

## Abstract

**Introduction:**

Curiosity toward the effects of psychedelic drugs on neural activation has increased due to their potential therapeutic benefits, particularly serotonergic psychedelics that act as 5-HT2A receptor agonists such as LSD, psilocybin, and MDMA. However, the pattern of their effects on neural activity in various brain regions in both clinical and healthy populations is still not well understood, and primary studies addressing this issue have sometimes generated inconsistent results.

**Objectives:**

The present meta-analysis aims to advance our understanding of the most widely used serotonergic psychedelics – LSD, psilocybin, and MDMA – by examining their effects on the functional activation throughout the whole brain among both clinical and healthy participants.

**Methods:**

We conducted this meta-analysis by applying multilevel kernel density analysis (MKDA) with ensemble thresholding to quantitatively combine existing functional magnetic resonance imaging (fMRI) studies that examined whole-brain functional activation of clinical or healthy participants who were administered a serotonergic psychedelic.

**Results:**

Serotonergic psychedelics, including LSD, psilocybin, and MDMA, exhibited significant effects (α=0.05) on neural activation in several regions throughout the cerebral cortex and basal ganglia, including effects that may be common across and unique within each drug.

**Conclusions:**

These observed effects of serotonergic psychedelics on neural activity advance our understanding of the functional neuroanatomy associated with their administration and may inform future studies of both their adverse and therapeutic effects, including emerging clinical applications for the treatment of several psychiatric disorders.

**Disclosure of Interest:**

None Declared

